# Long non-coding RNA DLEUI promotes papillary thyroid carcinoma progression by sponging miR-421 and increasing ROCK1 expression

**DOI:** 10.18632/aging.103642

**Published:** 2020-09-10

**Authors:** Rui Li, Taihu Wan, Jie Qu, Yang Yu, Ruipeng Zheng

**Affiliations:** 1 Department of Thyroid Surgery, The First Hospital of Jilin University, Changchun 130021, P.R. China; 2 Department of Division of Interventional Radiology, China-Japan Union Hospital of Jilin University, Changchun 130033, China; 3 Department of VIP Unit, China-Japan Union Hospital of Jilin University, Changchun 130033, China; 4 Department of General Surgery, China-Japan Union Hospital of Jilin University, Changchun 130033, China; 5 Department of Interventional Therapy, The First Hospital of Jilin University, Changchun 130021, P.R. China

**Keywords:** DLEU1, miR-421, papillary thyroid carcinoma, ROCK1

## Abstract

We investigated the role of long non-coding RNA DLEU1 (deleted in lymphocytic leukemia 1) in the progression of papillary thyroid carcinoma (PTC). DLEU1 levels were higher in PTC cell lines (BHP5-16, TPC-1,8505C, and SW1736) and patient tissues (n=54) than in a human thyroid follicular epithelial cell line (Nthy-ori3-1) or adjacent normal thyroid tissues. High DLEU1 expression correlated positively with lymph node metastasis and advanced clinical stages in PTC patients. Bioinformatics, dual luciferase reporter, and RNA pulldown assays confirmed that DLEU1 directly binds to miR-421. Moreover, bioinformatics and dual luciferase reporter assays showed that miR-421 directly binds to the 3’untranslated region of the rho-related coiled-coil kinase 1 (ROCK1) in TPC-1 cells. PTC patient tissues and cell lines showed high ROCK1 mRNA and protein levels as well as low miR-421 levels. CCK-8, flow cytometry, wound healing, and Transwell invasion assays demonstrated that DLEU1 silencing decreases TPC-1 cell proliferation, survival and progression, but they can be rescued by miR-421 knockdown or ROCK1 overexpression. DLEU1 knockdown in TPC-1 cells decreased *in vivo* xenograft tumor size and weight compared to controls in nude mice. These findings demonstrate that DLEU1 promotes PTC progression by sponging miR-421 and increasing ROCK1 expression.

## INTRODUCTION

Thyroid cancer is a predominant endocrine neoplasm whose incidence rates are rapidly increasing in the last few decades [1]. The prognosis for patients with papillary thyroid carcinoma (PTC) is highly favorable, but, distant metastases or local recurrence is reported in several cases [2]. The potential molecular mechanisms that regulate metastasis in PTC are still unclear [3]. Hence, elucidating the molecular mechanisms involved in PTC growth and progression are critical and urgent in order to discover effective diagnostic and prognostic biomarkers and novel therapeutic targets for PTC.

Long noncoding RNAs (lncRNAs) are a class of regulatory RNA molecules without any coding capacity that are longer than 200 nucleotides [4]. LncRNAs are widely reported to regulate tumor cell growth, migration, invasion, and apoptosis in several cancers [5–7]. Previous studies have identified some lncRNAs that regulate PTC progression by acting as tumor promoters or suppressors [8, 9]. Therefore, there is great potential for lncRNAs as novel therapeutic targets in PTC.

Deleted in lymphocytic leukemia 1 (DLEU1) is a lncRNA that is located on chromosome 13q14.3, and is aberrantly upregulated in several malignancies, including osteosarcoma, hepatocellular carcinoma, bladder cancer, glioma, cervical cancer, pancreatic ductal adenocarcinoma, colorectal cancer, non-small cell lung cancer, and oral gastric cancer [10]. Besides, LDEU1 promotes tumor cell proliferation, migration, apoptosis and invasion in several cancers [10]. However, the role of DLEU1 in PTC is not clear. Hence, in this study, we investigated the role of DLEU1 in PTC growth and progression using PTC cell lines and patient tissue samples. We also investigated the plausible mechanisms through which DLEU1 regulates PTC growth and progression.

## RESULTS

### DLEU1 expression is significantly upregulated in PTC cell lines and tissues

**Figure 1:**
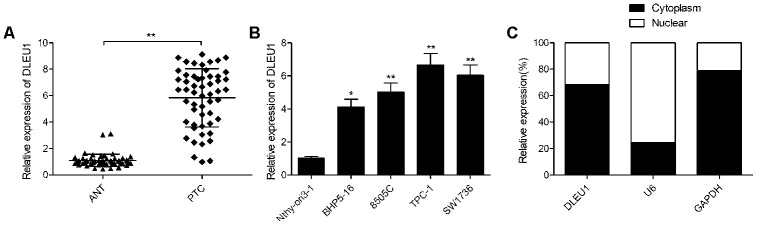
**DLEU1 is overexpressed in PTC tissues and cell lines.** (**A**) QRT-PCR analysis shows the expression of DLEU1 in 54 paired PTC tissues and adjacent normal thyroid tissues. (**B**) QRT-PCR analysis shows the expression of DLEU1 in four PTC cell lines (BHP5-16, 8505C, TPC-1, and SW1736) and the human thyroid follicular epithelial cells, Nthy-ori3-1. (**C**) QRT-PCR analysis shows the expression of DLEU1 in the cytoplasmic and nuclear extracts of TPC-1 cells. Note: The data are shown as the means ± SD of at least three independent experiments. *P< 0.05 and **P< 0.01.

Quantitative real time-PCR (qRT-PCR) analysis showed that DLEU1 expression was significantly higher in PTC tissues compared to matched adjacent noncancerous thyroid tissues from 54 PTC patients (Figure 1A). Next, we divided the 54 PTC patients into low (n=26) and high (n=28) DLEU expressing groups based on the mean DLEU1 expression in PTC tissues. Correlation analysis of DLEU1 expression and clinicopathological parameters showed that high DLEU1 expression was associated with lymph node metastasis and advanced TNM stages (Table 1). We then compared the expression of DLEU1 in human thyroid follicular epithelial cell line, Nthy-ori3-1 and four PTC cell lines, BHP5-16, TPC-1,8505C, and SW1736. QRT-PCR analysis showed higher DLEU1 expression in all PTC cell lines compared to the Nthy-ori 3-1 cells (Figure 1B). Furthermore, qRT-PCR analysis showed that DLEU1 expression was significantly higher in the cytoplasm compared to the nucleus in the TPC-1 cells (Figure 1C).

**Table 1:** Correlation between clinicopathological parameters and DLEU1 expression in PTC tissues.

**Variables**	**No. of cases**	**DLEU1 expression**		***P* value**
		**High**	**Low**	
**Age (years)**				*P*=0.5804
**<60**	22	10	12	
**≥60**	32	18	14	
**Gender**				*P*=0.5743
**Male**	20	9	11	
**Female**	34	19	15	
**TNM stage**				*P*=0.0099
**T1-T2**	41	17	24	
**T3-T4**	13	11	2	
**Tumor size(cm)**				*P*=0.5859
**<1**	33	16	17	
**≥1**	21	12	9	
**Lymph node metastasis**				*P*=0.0020
**No**	39	15	24	
**Yes**	15	13	2	

### DLEU1 knockdown inhibits PTC cell proliferation, migration and invasion

**Figure 2:**
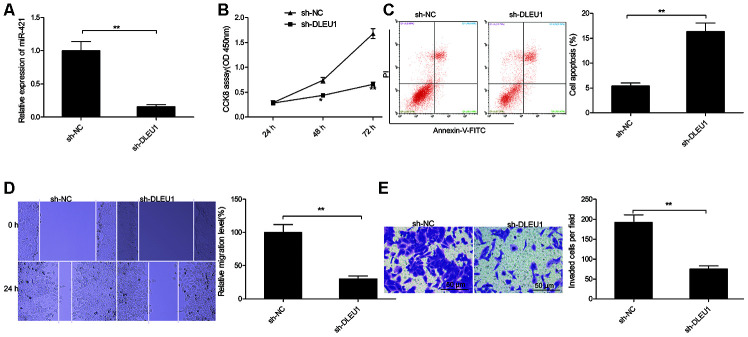
**DLEU1 knockdown inhibits proliferation, migration and invasion of TPC-1 cells.** (**A**) QRT-PCR analysis shows DLEU1 levels in sh-NC- and sh-DLEU1-transfected TPC-1 cells. (**B**) CCK8 assay results show proliferation rates of sh-NC- and sh-DLEU1-transfected TPC-1 cells. (**C**) Flow cytometry analysis shows the percentage apoptosis in sh-NC- and sh-DLEU1-transfected TPC-1 cells based on Annexin-V staining. (**D**) Wound healing assay results show the migration efficiency of sh-NC- and sh-DLEU1-transfected TPC-1 cells. (**E**) Transwell invasion assay results show the invasiveness of sh-NC- and sh-DLEU1-transfected TPC-1 cells. Note: The data is represented as the means ± SD of at least three independent experiments. *P< 0.05 and **P< 0.01.

We then analyzed the role of DLEU1 in tumorigenesis by evaluating the effects of DLEU1 knockdown on *in vitro* proliferation, migration and invasion of PTC cell lines. QRT-PCR analysis showed that DLEU1 levels were significantly reduced in sh-DLEU1-transfected TPC-1 cells compared to the sh-NC-transfected TPC-1 cells (Figure 2A). CCK8 assay results showed that proliferation was significantly reduced in the DLEU1-knockdown TPC-1 cells compared to the controls (Figure 2B). Furthermore, apoptosis was significantly increased in the DLEU1-knockdown TPC-1 cells compared to the controls (Figure 2C). Moreover, wound healing assay showed that cell migration was significantly reduced in DLEU1-knockdown TPC-1 cells compared to the controls (Figure 2D). Transwell invasion assay showed that invasiveness of DLEU1-knockdown TPC-1 cells was significantly reduced compared to the controls (Figure 2E).

### DLEU1 sponges miR-421 in PTC cells

**Figure 3:**
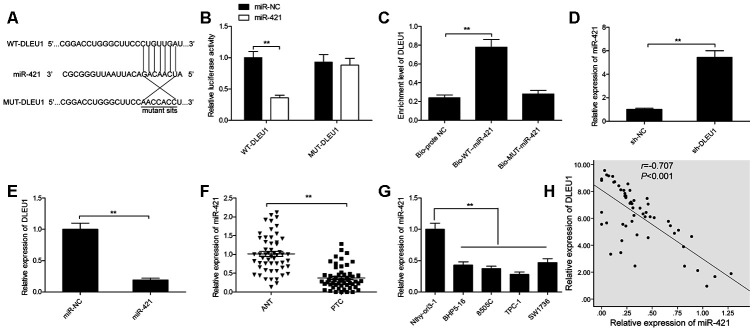
**DLEU1 sponges miR-421 in PTC cells.** (**A**) The diagram shows the predicted miR-421 binding sites in the 3’UTR of DLEU1 and the mutations in the miR-421 binding sites. (**B**) Dual luciferase reporter assay shows the relative luciferase activity of TPC-1 cells co-transfected with miR-421 mimic or miR-NC plus luciferase reporter plasmid with the wild-type DLEU1 (WT-DLEU1) or mutant DLEU1 (MUT-DLEU1). The miR-421 binding sites are mutated in the mutant DLEU1. (**C**) QRT-PCR results show DLEU1 levels in the RNA pull down extracts using biotinylated wild-type or mutant miR-421. (**D**) QRT-PCR analysis shows miR-421 levels in control and DLEU1-silenced TPC-1 cells. (**E**) QRT-PCR analysis shows DLEU1 levels in control and miR-421 mimic-transfected TPC-1 cells. (**F**) QRT-PCR analysis shows miR-421 expression in 54 paired PTC and adjacent normal thyroid tissues.(**G**) QRT-PCR analysis shows the expression of miR-421 in four PTC cell lines (BHP5-16, 8505C, TPC-1, and SW1736) and the human thyroid follicular epithelial cell line, Nthy-ori3-1. (**H**) Spearman’s correlation analysis shows that DLEU1 expression is inversely related to miR-421 expression in PTC tissues (n=54). Note: The data are represented as the means ± SD of at least three independent experiments. *P< 0.05 and **P< 0.01.

Next, we investigated potential DLEU1-binding miRNAs using the Starbase 2.0 online software and identified miR-421 as a potential candidate. Previous reports show that miR-421 is involved in the initiation and progression of multiple cancers, such as glioma, breast cancer, osteosarcoma and gastric cancer [11–14]. The sequence of miR-421 and the putative DLEU1- binding sites are shown in Figure 3A. Dual luciferase reporter assay showed that high luciferase activity was found in TPC-1 cells co-transfected with miR-421 mimics and the wild-type DLEU1 luciferase reporter (WT-DLEU1), but was absent in TPC-1 cells transfected with miR-421 mimics and the mutant DLEU1 luciferase reporter (MUT-DLEU1) as shown in Figure 3B. RNA pull-down assay results showed that miR-421 DLEU1 directly binds to miR-421 in TPC-1 cells (Figure 3C). Moreover, miR-421 levels were significantly higher in DLEU1-knockdown TPC-1 cells compared to the controls (Figure 3D). The miR-421-mimic-transfected TPC-1 cells showed significantly lower levels of DLEU1 compared to the controls (Figure 3E). Furthermore, miR-421 levels were significantly lower in PTC tissues compared to the adjacent normal thyroid tissues in 54 PTC patients (Figure 3F). Moreover, miR-421 expression was downregulated in all 4 PTC cell lines (BHP5-16, 8505C, TPC-1, and SW1736) compared to the Nthy-ori3-1 cells (Figure 3G). Besides, DLEU1 expression showed inverse correlation with miR-421 expression in PTC tissues (Figure 3H). These results demonstrate that DLEU1 directly binds and downregulates miR-421 in PTC cells and tissues.

### ROCK1 is a direct target of miR-421 in PTC cells

**Figure 4:**
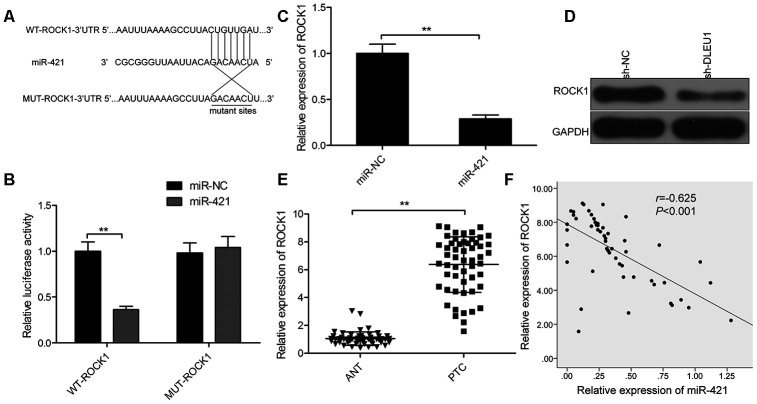
**ROCK1 is a direct target of miR-421 in PTC cells.** (**A**) The predicted miR-421 binding sites in the 3’UTR of ROCK1 and the mutated sequence are shown. (**B**) Dual luciferase reporter assay shows the relative luciferase activity in TPC-1 cells co-transfected with miR-421 mimic or miR-NC and luciferase reporter plasmid with wild-type ROCK1-3’-UTR (WT-ROCK1) or mutant ROCK1-3’-UTR (MUT-ROCK1). (**C**) QRT-PCR analysis shows the ROCK1 mRNA levels in miR-NC- and miR-421 mimic-transfected TPC-1 cells. (**D**) Western blot analysis shows ROCK1 protein levels in miR-NC- and miR-421 mimic-transfected TPC-1 cells. (**E**) QRT-PCR analysis shows *ROCK1* mRNA expression in 54 paired PTC and adjacent normal thyroid tissues. (**F**) Spearman correlation analysis shows that ROCK1 expression is inversely related to miR-421 expression in PTC tissues (n=54). Note: The data are represented as the means ± SD of at least three independent experiments. *P< 0.05 and **P< 0.01.

Next, we searched for the target genes of miR-421 using the TargetScan software program and identified ROCK1-3’UTR as a potential miR-421 target (Figure 4A). Furthermore, dual luciferase reporter assay showed that luciferase activity was significantly decreased in TPC-1 cells co-transfected with reporter vector containing the wild-type ROCK1 3'-UTR (WT-ROCK1) and miR-421 mimics, but was not affected in TPC-1 cells transfected with a luciferase reporter vector with mutant ROCK1 3'-UTR (MUT-ROCK1) and miR-421 mimics (Figure 4B). Moreover, miR-421 overexpression significantly downregulated ROCK1 mRNA and protein levels in TPC-1 cells (Figure 4C and 4D). Besides, qRT-PCR analysis showed that ROCK1 mRNA levels were significantly higher in PTC tissues compared to adjacent normal thyroid tissues (Figure 4E). In PTC tissues, miR-421 expression negatively correlated with ROCK1 mRNA levels (Figure 4F, *r*=-0.625, *P*<0.001). These results confirm that miR-421 binds directly to the 3’UTR of ROCK1 in TPC-1 cells.

### DLEU1 modulates miR-421/ROCK1 axis in PTC cells

**Figure 5:**
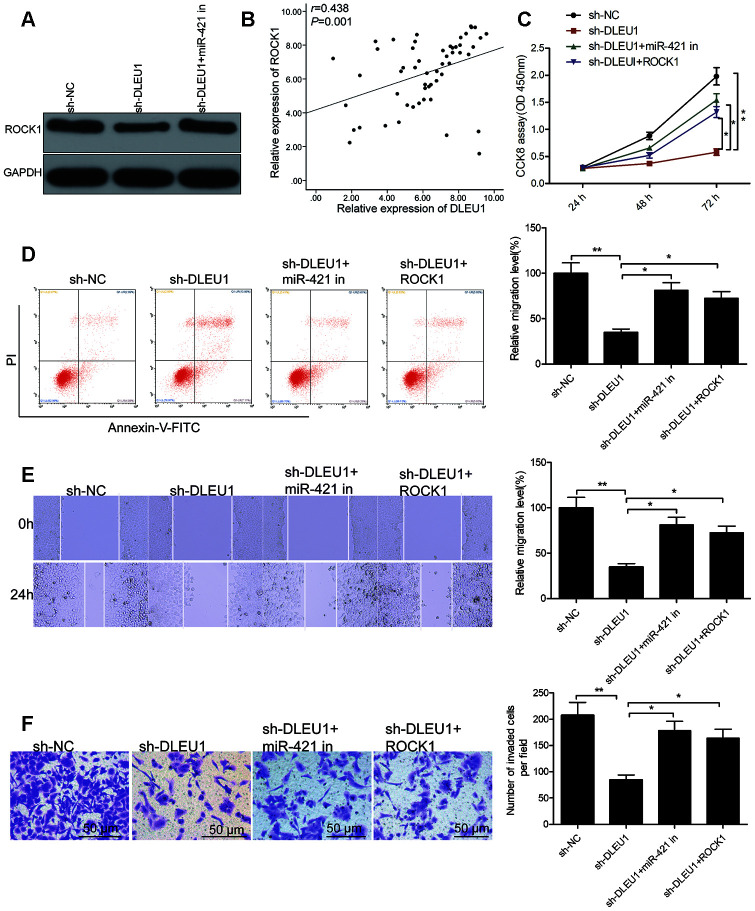
**DLEU1 regulates PTC cell growth and progression through the miR-421/ROCK1 axis.** (**A**) Western blot analysis shows ROCK1 protein levels in sh-NC-, sh-DLEU1- and sh-DLEU1 plus miR-421 inhibitor-transfected TPC-1 cells. (**B**) Spearman correlation analysis shows that ROCK1 mRNA expression is inversely related to DLEU1 expression in PTC tissues (n=54). (**C**) CCK-8 assay analysis shows proliferation rates of TPC-1 cells transfected with sh-NC, sh-DLEU1, sh-DLEU1 plus miR-421 inhibitor, and sh-DLEU1 plus ROCK1 overexpression plasmid. (**D**) Flow cytometry analysis shows percentage apoptosis (% Annexin-V^+^ cells) in TPC-1 cells transfected with sh-NC, sh-DLEU1, sh-DLEU1 plus miR-421 inhibitor, and sh-DLEU1 plus ROCK1 overexpression plasmid. (**E**) Wound healing assay results show the numbers of migrating cells in the TPC-1 cells transfected with sh-NC, sh-DLEU1, sh-DLEU1 plus miR-421 inhibitor, and sh-DLEU1 plus ROCK1 overexpression plasmid. (**F**) Transwell invasion assay results show the numbers of invading cells in the TPC-1 cells transfected with sh-NC, sh-DLEU1, sh-DLEU1 plus miR-421 inhibitor, and sh-DLEU1 plus ROCK1 overexpression plasmid. Note: The data is represented as the means ± SD of at least three independent experiments. *P< 0.05 and **P< 0.01.

We further investigated the association between DLEU1, miR-421 and ROCK1 in TPC-1 cells. Western blot analysis showed that ROCK1 protein levels were significantly reduced in sh-DLEU1-transfected TPC1 cells, but was reversed by co-transfection with the miR-421 inhibitor (Figure 5A). Moreover, DLEU1 levels showed positive correlation with ROCK1 in PTC tissues (Figure 5B). These results suggest that DLEU1 modulates the expression of ROCK1 by sponging miR-421 in PTC tissues and cells.

Next, we analyzed if DLEU1 exerts its oncogenic effects in PTC via the miR-421/ROCK1 axis. CCK-8 assay results showed that proliferation was significantly reduced in DLEU1-silenced TPC-1 cells compared to the controls, but was partially rescued by overexpression of ROCK1 or miR-421 knockdown (Figure 5C). Furthermore, flow cytometry analysis demonstrated that apoptosis was significantly higher in DLEU1 knockdown TPC-1 cells, but partially reversed by the overexpression of ROCK1 or miR-421 knockdown (Figure 5D) Transwell invasion assay showed that invasiveness of DLEU1 knockdown TPC-1 cells was significantly lower than the controls, but was partially rescued by ROCK1 overexpression and miR-421 knockdown (Figure 5E and 5F). Collectively, these results suggest that DLEU1 promotes PTC cell proliferation and invasiveness via miR-421/ROCK1 axis.

### DLEU1 knockdown reduces *in vivo* xenograft PTC tumor growth

**Figure 6:**
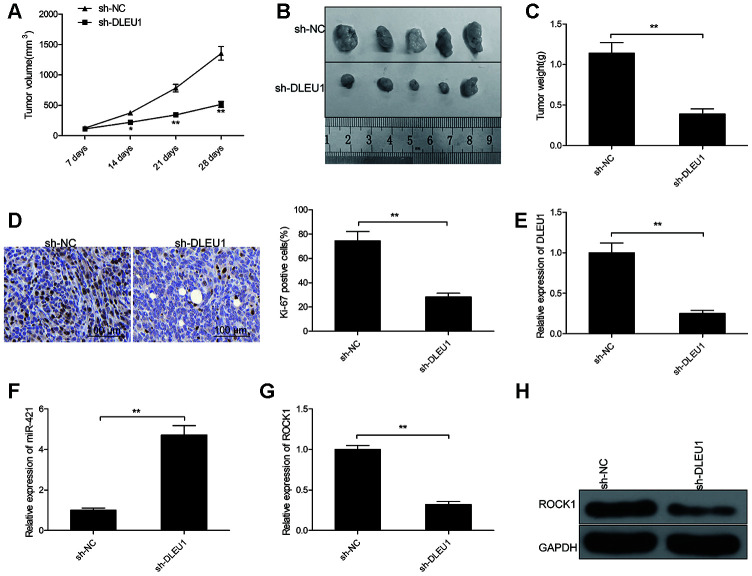
**DLEU1 knockdown reduces *in vivo* growth of xenograft tumors in nude mice model.** (**A**) The curve shows the rate of growth of xenograft tumors generated from subcutaneously injected control and DLEU1 knockdown TPC-1 cells in the nude mice (n=5 each). Tumor growth was measured every 7 days for 28 days. (**B**) The representative images show the xenograft tumors in nude mice that are subcutaneously injected with control and DLEU1 knockdown TPC-1 cells for 28 days. (**C**) The histogram plot shows the weight of xenograft tumors derived from nude mice subcutaneously injected with control and DLEU1 knockdown TPC-1 cells. (**D**) Representative images show IHC staining with the anti-Ki67 antibody of xenograft tumor tissue sections derived from nude mice subcutaneously injected with control and DLEU1 knockdown TPC-1 cells. (**E**–**F**) QRT-PCR analysis shows the levels of DLEU1 and miR-421 in the xenograft tumor tissues derived from nude mice subcutaneously injected with control and DLEU1 knockdown TPC-1 cells. (**G**) QRT-PCR analysis shows ROCK1 mRNA levels in the xenograft tumor tissues derived from nude mice subcutaneously injected with control and DLEU1 knockdown TPC-1 cells. (**H**) Western blot analysis shows ROCK1 protein levels in the xenograft tumor tissues derived from nude mice subcutaneously injected with control and DLEU1 knockdown TPC-1 cells. Note: The data are represented as the means ± SD of at least three independent experiments. *P< 0.05 and **P< 0.01.

Finally, we examined the *in vivo* effects of DLEU1 by establishing a xenograft tumor model through subcutaneous injection of control or DLEU1-knockdown TPC1 cells into nude mice. The tumor growth was significantly reduced in the sh-DLEU1 group compared to the sh-NC group (Figure 6A). The size and weight of xenograft tumors was significantly smaller in the sh-DLEU1 group compared to the sh-NC group (Figure 6B and 6C). IHC analysis of xenograft tumor tissue specimens showed that Ki-67- positive cells were significantly decreased in the sh-DLEU1 group tumors compared to those from the sh-NC group (Figure 6D). QRT-PCR analysis showed that DLEU1 and ROCK1 mRNA levels were reduced and miR-421 levels were higher in the sh-DLEU1 group tumors compared to the sh-NC group tumors (Figure 6E–6G). Western blot analysis showed that ROCK1 protein levels were reduced in the sh-DLEU1 group tumors compared to the sh-NC group tumors (Figure 6H).

## DISCUSSION

Several lncRNAs act as tumor suppressors or oncogenes and regulate PTC growth and progression [8, 9]. Yin et al. demonstrated that lncRNA HOXA11-AS sponged miR-761, which suppresses PTC progression by targeting TRIM29 [15]. Tong et al. demonstrated that ZFAS1 promotes PTC progression by sponging miR-590-3p and upregulating HMGA2 [16]. Zhuang et al. showed that lncRNA ABHD11-AS1 promotes PTC progression by regulating the miR-199a-5p/SLC1A5 axis [17]. In this study, we demonstrate that DLEU1 promotes PTC progression by regulating the miR-421/ROCK1 axis.

DLEU1 is upregulated in several cancers, including non-small cell lung cancer [18], gastric cancer, clear cell renal cell carcinoma [19], glioma [20], bladder cancer [21], hepatocellular carcinoma [22], breast cancer [23], colorectal cancer [24], ovarian cancer [25] and cervical cancer [26]. While DLEU1 acts as an oncogene in these cancers, its regulatory mechanism and biological role in PTC is not clear. In the current study, we demonstrate that DLEU1 is up-regulated in both PTC cell lines and tissues. Furthermore, high DLEU1 levels correlate with poor prognosis of PTC patients. DLEU1 knockdown suppresses *in vitro* PTC cell proliferation and invasion, and inhibits xenograft tumor growth in the nude model mice. These results suggest that DLEU1 plays an oncogenic role in PTC.

LncRNAs act as competing endogenous RNAs and regulate gene transcription by sponging miRNAs and inhibiting their ability to bind to their target mRNAs [27, 28]. Previous studies show that DLEUI acts as a ceRNA for miR-4429 [29], miR-671-5p [30], miR-133a [22], miR-99b [21], miR-300 [23] and miR-490-3p [24]. We used the Starbase 2.0 software to search for DLEU1-binding miRNAs and identified miR-421 as a potential target of DLEU1. Previous reports demonstrate that miR-421 regulates the growth and progression of glioma, breast cancer, osteosarcoma and gastric cancer [11–14]. In our study, dual luciferase and RNA pull down assays, and DLEU1 knockdown experiments confirmed that DLEU1 binds directly to miR-421and inhibit its expression in PTC cells. Moreover, we demonstrate that miR-421 expression negatively correlates with DLEU1 levels in the PTC tissues. Furthermore, DLEU1-knockdown TPC-1 cells show increased apoptosis and decreased proliferation and migration, but these effects are partially rescued by inhibiting miR-421. These data suggest that DLEU1 promotes growth and progression of PTC by inhibiting miR-421 expression and function.

We also demonstrate that MiR-421 inhibits ROCK1 expression by directly binding to the 3′-UTR of ROCK1 in PTC cells. ROCK1 functions as an oncogene in many cancer types, such as non-small lung cancer, breast cancer, ovarian cancer and prostate cancer [31, 32]. ROCK1 is overexpressed in PTC tissues and promotes PTC cell proliferation and invasion [33, 34]. Previous reports also show that miR-26a [35], miR-584 [36] and miR-150 [37] directly target ROCK1 in PTC. We demonstrate that knockdown of DLEU1 decreases ROCK1 expression in TPC-1 cells, but, this effect is reversed by co-transfection with the miR-421 inhibitor. Moreover, ROCK1 expression negatively correlates with miR-421 levels and positive correlates with DLEU1 levels in PTC tissues. ROCK1 overexpression partially rescues the reduced proliferation, migration and invasion of DLEU1-knockdown TPC-1 cells. These results demonstrate that DLEUI exerts its oncogenic role in PTC by regulating the miR-421/ROCK1 axis.

In conclusion, our study demonstrates that DLEU1 promotes PTC growth and progression by regulating the miR-421/ROCK1 axis. Our data suggests that DLEU1 is a potential therapeutic target for PTC.

## MATERIALS AND METHODS

### PTC patient tissue samples

We obtained fresh paired PTC and adjacent non-tumor thyroid tissues from 54 PTC patients who underwent surgery at our hospital between March 2017 and March 2018. The samples were verified by pathological examination and stored at -80 °C until further use in experiments. These 54 patients did not receive any anti-tumor treatments before surgery. We obtained signed informed consent from all patients. The study was approved by the Ethics committee of the Jilin University.

### PTC cell lines and transfection protocols

We purchased 4 human PTC cell lines, BHP5-16,8505C, TPC-1, SW1736, and Nthy-ori3-1from the Chinese Academy of Sciences (Beijing, China). The cell lines were culturedin DMEM medium (Gibco, CA, USA) supplemented with heat-inactivated 10% fetal bovine serum (FBS, Gibco) and antibiotics (100 U/mL penicillin and 100 mg/mL streptomycin) (Gibco) in a incubator with 5% CO_2_ at 37°C. The shRNA against DLEU1 (sh-DLEU1) and the negative control shRNA (sh-NC) were designed and synthesized by Shanghai Gene Pharma (Shanghai, China), and cloned into the pHBLV-U6-ZSGreen-puro lentivirus vector (HANBIO, Shanghai, China). Then, we infected the TPC-1 cells with lentiviral particles containing the shRNAs and selected the transfected cells by growing them in medium containing 1 μg/ml puromycin (Sigma, USA) for 7 days. The negative control mimics (miR-NC) and Hsa-miR-421 mimics (miR-421) were purchased from RiboBio (Guangzhou, China). The ROCK1 overexpressing plasmid vector (pCDNA3.1-ROCK1) was purchased from Western Biotechnology (Chongqing, China). The TPC-1 cells were transfected at 50%–70% confluence with pCDNA3.1 control vector, pCDNA3.1-ROCK1, miR-NC or miR-421 using Lipofectamine 2000 (Invitrogen, USA) according to the manufacturer’s recommendations.

### Quantitative real-time PCR (qRT-PCR)

We extracted total cellular RNA and miRNA from cultured cells or tumor tissue sections using the TRIzol reagent (Invitrogen, Carlsbad, CA, USA) and miRNA Isolation Kit (Qiangen, German), respectively. The miR-421 expression was examined as described previously [11]. To analyze the levels of DELU1 and *ROCK1* mRNA, cDNA was synthesized from total cellular or tissue RNA. Then, qPCR analysis was performed using the PrimeScript RT Reagent Kit (Takara). GAPDH and U6 were used as internal controls for DLEU1 or ROCK1 and miR-421, respectively. The qPCR primers for DLEU1, ROCK1 and miR-421 were as previously described [18, 11, 34]. The relative expression of DLEU1, ROCK1 mRNA and miR-421 was calculated using the 2^–ΔΔCt^ method.

To determine DLEU1 levels in the cytoplasm and nuclei, we isolated cytoplasm and the nuclei using the Nuclear/Cytosol Fractionation Kit (Biovision, San Francisco, CA, USA) according to manufacturer’s instructions. Then, we extracted the total RNA from the cytoplasmic and the nuclear extracts and quantified DLEU1 by qRT-PCR using U6 and GAPDH as internal controls for nucleus and cytoplasm, respectively.

### CCK-8 cell proliferation assay

CCK-8 assay was performed to determine the proliferation of different experimental groups of TPC-1 cells. Briefly, the transfected TPC-1 cells (10^4^ cells / well) in RPMI 1640 with 10% FBS were seeded into 96-well plates and grown for 24 h in a humidified chamber at 37°C and 5% CO2. Then, 10 μl CCK-8 solution was added to each well at different time points (24 h, 48 h and 72 h) and the cells were further incubated for another 4 h. Then, the optical density (OD) was measured at 450 nm using a microplate reader (Bio-Rad Laboratories, Hercules, CA, USA) to determine the rate of proliferation.

### Flow cytometry analysis of apoptosis analysis

We performed flow cytometry analysis to determine apoptotic rate of different experimental groups of TPC-1 cells using the Annexin V/Dead Cell Apoptosis Kit (Invitrogen) according to manufacturer’s instructions. The stained cells were analyzed using a MACSQuant^TM^ Flow Cytometer (Miltenyi Biotec, USA) and the percentages of apoptotic (Annexin-V^+^) cells in each sample was determined using the Cell Quest software version 3.2 (BD Biosciences, USA).

### Wound healing assay

To determine the role of miR-421 on tumor cell migration, we performed wound healing assay by seeding 5.0 × 10^4^ transfected TPC-1 cells per well in 6-well plates. When the cultures reached confluence, an area of cells was wounded with a pipette tip. Then, after rinsing with PBS, the cells were further incubated in serum-free medium for 24 h. Images were captured under a light microscope(Olympus, Tokyo, Japan) at 0 and 24 h to determine the extent of wound healing. The wounded area was measured using the Image J software 3.0.

### Transwell invasion assay

We performed Transwell invasion assay to determine the role of miR-421 on tumor cell invasiveness. Briefly, transfected TPC-1 cells were seeded in the upper chamber of the BD BioCoat™Matrigel invasion chambers (BD Biosciences) in serum-free DMEM medium. In the lower chamber, 600 μl of DMEM medium plus 10% FBS was added as a chemo attractant. The Transwell chambers were incubated for 24 h in a humidified chamber at 37°C and 5% CO_2_. Then, the cells that invaded onto the lower side of the Transwell membrane between the upper and lower chambers were fixed, stained with crystal violet solution, and counted in 6 randomly chosen fields using a X71Olympus light microscope.

### Dual luciferase reporter assays

We cloned the wild-type (WT) or mutant (MUT) miR-421 binding sequences in the 3’UTR of ROCK1 (WT-ROCK1 and MUT-ROCK1) and DLEU1 (WT-DLEU1 and MUT-DLEU1) into the pGL3-promoter vector. The TPC-1 cells were co-transfected with the luciferase reporter vectors carrying different constructs and the miR-421 mimics or negative control mimics using the lipofectamine 2000 reagent (Invitrogen) according to manufacturer’s instructions. Luciferase activity was measured after 48 h using the Dual Luciferase Assay Kit (Promega, WI, USA) according to the manufacturer’s instructions.

### RNA pull-down assays

For the RNA pull-down assay, TPC-1 cells were transfected with biotinylated wild type (WT) miR-421(Bio-WT-miR-421), mutant (MUT) miR-421(Bio-MUT-miR-421) and negative control (Bio-miR-NC) (Guangzhou RiboBio Co., Ltd).The cell lysates were collected at 24 h after transfection and incubated with Dynabeads M-280 Streptavidin (Invitrogen) for 3 h at 4 °C fellowing the manufacturer’s protocol. Finally, DLEU1 levels were estimated in the RNA pull down extract using qRT-PCR.

### Western blotting

Total cellular or tissue protein lysates were prepared using the RIPA lysis buffer with 1% protease inhibition from Sigma-Aldrich (Darmstadt, Germany) and quantified. Then, 20 μg of total protein lysates from each specimen was separated on a 10% SDS-PAGE and transferred using the semi-dry transfer method onto PVDF membranes (Billerica). Then, the membranes were blocked with 5% skimmed milk in TBST at room temperature for 1 h. The membranes were incubated overnight at 4°C with primary antibodies against ROCK1 and GAPDH (Santa Cruz Biotechnology, CA, USA), followed by incubation at room temperature for 1 h with HRP-conjugated secondary antibodies((Santa Cruz Biotechnology). The protein blots were developed using the ECL (Enhanced Chemiluminescence) Detection Kit (BOSTER, USA). ROCK1 levels relative to GAPDH1 were quantified using the Image J software.

### Xenograft tumor model

The animal experiments were performed in accordance with the protocol approved by the Institutional Animal Care and Use Committee review board of Jilin University. We subcutaneously injected 5 × 10^6^ stably transfected sh-NC- or sh-DLEU1-transfected TPC1 cells into the left flanks of 5-6 week old female nude mice (18-25g; n=5). Tumor volume (V) was measured once a week using the following formula: V = (Length × Width^2^)/2. Then, after 28 days, the mice were euthanized. The tumors were harvested, weighed, and stored for qRT-PCR and immunohistochemistry (IHC).

### Immunohistochemistry (IHC)

IHC was performed to detect Ki-67 expression in the xenograft tumors as described previously [38]. The primary rabbit anti-Ki-67 antibody and the secondary goat anti-rabbit IgG were purchased from Abcam (Cambridge, UK). Ki-67 expression was quantified using the Image-pro-plus (IPP) software (Media Cybernetics, Washington, USA).

### Statistical analysis

The data is shown as the means ± SD (standard deviation) from at least three independent experiments. Statistical analysis was performed using the SPSS software version 19.0 (IBM SPSS, Armonk, NY, USA). The continuous data was compared using the Student’s two-tailed t-test for 2 groups and one-way analysis of variance (ANOVA) for more than 2 groups. Spearman correlation analysis was used to determine the relationship between DLEU1, miR-421 and ROCK1 expression. The Chi–square test was used to analyze the correlation between DLEU1 expression and the clinicopathological parameters of PTC patients. *P*<0.05 was considered statistically significant.
