# Correction for: Long non-coding RNA DLEUI promotes papillary thyroid carcinoma progression by sponging miR-421 and increasing ROCK1 expression

**DOI:** 10.18632/aging.204506

**Published:** 2023-01-31

**Authors:** Rui Li, Taihu Wan, Jie Qu, Yang Yu, Ruipeng Zheng

**Affiliations:** 1 Department of Thyroid Surgery, The First Hospital of Jilin University, Changchun 130021, P.R. China; 2 Department of Division of Interventional Radiology, China-Japan Union Hospital of Jilin University, Changchun 130033, China; 3 Department of VIP Unit, China-Japan Union Hospital of Jilin University, Changchun 130033, China; 4 Department of General Surgery, China-Japan Union Hospital of Jilin University, Changchun 130033, China; 5 Department of Interventional Therapy, The First Hospital of Jilin University, Changchun 130021, P.R. China

**Keywords:** DLEU1, miR-421, papillary thyroid carcinoma, ROCK1

**This article has been corrected:** The authors found that **Figure 5** contains the same graph panel for “Cell apoptosis (%)” in **Figures 5D** and **5E.** They replaced incorrect graph in **Figure 5E** with the panel corresponding to the wound healing assay graph measuring “Relative migration level (%)” from the original sets of experiments. This correction has no impact on the experimental outcome or conclusions.

Corrected **Figure 5** is presented below.

**Figure 5:**
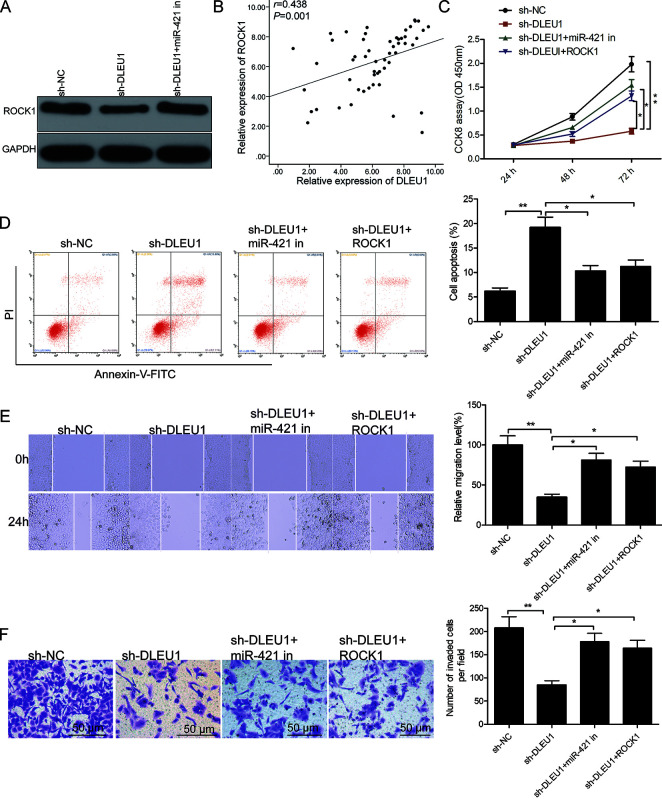
**DLEU1 regulates PTC cell growth and progression through the miR-421/ROCK1 axis.** (**A**) Western blot analysis shows ROCK1 protein levels in sh-NC-, sh-DLEU1- and sh-DLEU1 plus miR-421 inhibitor-transfected TPC-1 cells. (**B**) Spearman correlation analysis shows that ROCK1 mRNA expression is inversely related to DLEU1 expression in PTC tissues (n=54). (**C**) CCK-8 assay analysis shows proliferation rates of TPC-1 cells transfected with sh-NC, sh-DLEU1, sh-DLEU1 plus miR-421 inhibitor, and sh-DLEU1 plus ROCK1 overexpression plasmid. (**D**) Flow cytometry analysis shows percentage apoptosis (% Annexin-V^+^ cells) in TPC-1 cells transfected with sh-NC, sh-DLEU1, sh-DLEU1 plus miR-421 inhibitor, and sh-DLEU1 plus ROCK1 overexpression plasmid. (**E**) Wound healing assay results show the numbers of migrating cells in the TPC-1 cells transfected with sh-NC, sh-DLEU1, sh-DLEU1 plus miR-421 inhibitor, and sh-DLEU1 plus ROCK1 overexpression plasmid. (**F**) Transwell invasion assay results show the numbers of invading cells in the TPC-1 cells transfected with sh-NC, sh-DLEU1, sh-DLEU1 plus miR-421 inhibitor, and sh-DLEU1 plus ROCK1 overexpression plasmid. Note: The data is represented as the means ± SD of at least three independent experiments. *P< 0.05 and **P< 0.01.

